# HDL Cholesterol Efflux and Serum Cholesterol Loading Capacity Alterations Associate to Macrophage Cholesterol Accumulation in FH Patients with Achilles Tendon Xanthoma

**DOI:** 10.3390/ijms23158255

**Published:** 2022-07-26

**Authors:** Maria Pia Adorni, Marta Biolo, Francesca Zimetti, Marcella Palumbo, Nicoletta Ronda, Paolo Scarinzi, Paolo Simioni, Maria Giovanna Lupo, Nicola Ferri, Lorenzo Previato, Franco Bernini, Alberto Zambon

**Affiliations:** 1 Unit of Neuroscience, Department of Medicine and Surgery, University of Parma, 43125 Parma, Italy; mariapia.adorni@unipr.it; 2 Department of Medicine, University of Padua, 35128 Padua, Italy; marta.biolo@gmail.com (M.B.); scarinzi.paolo@gmail.com (P.S.); paolo.simioni@unipd.it (P.S.); mariagiovanna.lupo@unipd.it (M.G.L.); nicola.ferri@unipd.it (N.F.); lorenzo.previato@unipd.it (L.P.); 3 Department of Food and Drug, University of Parma, 43124 Parma, Italy; francesca.zimetti@unipr.it (F.Z.); marcella.palumbo@unipr.it (M.P.); nicoletta.ronda@unipr.it (N.R.); 4 IRCCS MultiMedica, 20099 Milano, Italy; alberto.zambon@unipd.it

**Keywords:** Achilles tendon, xanthoma, macrophage, HDL cholesterol efflux, serum cholesterol loading

## Abstract

Achilles tendon xanthoma (ATX) formation involves macrophage cholesterol accumulation within the tendon, similar to that occurring in atheroma. Macrophage cholesterol homeostasis depends on serum lipoprotein functions, namely the high-density lipoprotein (HDL) capacity to promote cell cholesterol efflux (cholesterol efflux capacity, CEC) and the serum cholesterol loading capacity (CLC). We explored the HDL-CEC and serum CLC, comparing 16 FH patients with ATX to 29 FH patients without ATX. HDL-CEC through the main efflux mechanisms mediated by the transporters ATP binding cassette G1 (ABCG1) and A1 (ABCA1) and the aqueous diffusion (AD) process was determined by a cell-based radioisotopic technique and serum CLC fluorimetrically. Between the two groups, no significant differences were found in terms of plasma lipid profile. A trend toward reduction of cholesterol efflux via AD and a significant increase in ABCA1-mediated HDL-CEC (+18.6%) was observed in ATX compared to no ATX patients. In ATX-presenting patients, ABCG1-mediated HDL-CEC was lower (−11%) and serum CLC was higher (+14%) compared to patients without ATX. Considering all the patients together, ABCG1 HDL-CEC and serum CLC correlated with ATX thickness inversely (*p* = 0.013) and directly (*p* < 0.0001), respectively. In conclusion, lipoprotein dysfunctions seem to be involved in ATX physiopathology and progression in FH patients.

## 1. Introduction

Familial hypercholesterolemia (FH) is defined as an inherited autosomal codominant genetic disease, characterized by elevated plasma low-density lipoprotein (LDL) cholesterol (LDL-c) and a high risk of premature cardiovascular disease (CVD) [1,2]. Patients with homozygous FH indeed develop severe, premature, atherosclerotic CVD often before 20 years of age, while heterozygous FH patients present a clinically relevant atherosclerotic cardiovascular disease in early middle age [3]. High LDL-c levels lead to the development of cholesterol deposits not only in the arteries, favoring atheroma formation, but also in the skin and tendons, generating xanthomata. Achilles tendons are the most common sites of xanthoma formation. Achilles tendon xanthomas (ATX) are considered pathognomonic features of FH, so their presence is included in the Dutch Lipid Clinic Network (DLCN) score, the diagnostic algorithm most widely used in the clinical setting for FH diagnosis [4]. The ability to detect ATX by physical examination is limited; thus, in the last years, many studies have evaluated the diagnostic performance of imaging techniques such as tendon ultrasound or magnetic resonance imaging (MRI). Evidence suggests that Achilles tendon ultrasonography improves clinicians’ accuracy in identifying ATX that is not physically evident [5].

The presence of ATX provides the opportunity for an early definitive diagnosis of FH and a timely optimization of effective lipid-lowering therapy. Early treatment indeed can reduce LDL-c burden, resulting in attenuation of atherosclerotic disease progression and improved cardiovascular (CV) outcome [2,6]. 

Moreover, xanthomas could represent a tool to better assess the cardiovascular risk of FH patients. Tendon xanthomas are associated with other CV risk factors, such as age, smoking, arterial hypertension, and premature CVD, suggesting that their detection represents an early marker for more aggressive lipid-lowering intervention [7,8].

The presence of ATX and the genetic status of FH are independently as well as additively associated with an increased coronary artery disease (CAD) risk [9]. Furthermore, evidence shows that patients with ATX demonstrate more severe CAD [8,10], suggesting a close pathophysiological relationship. In this regard, similarly to atheroma in the arterial wall, ATX is a consequence of disturbances in cholesterol handling [11]. Cholesterol deposition in atheroma and peripheral tissues occurs mainly in macrophages and derives from an imbalance between cholesterol uptake from LDL or their modified forms and cholesterol efflux to high-density lipoproteins (HDL) [12]. Thus, in the case of xanthoma, LDL derived from the circulation accumulate into tendons, and subsequently, modified LDL is actively uptaken by macrophages [13] with a process similar to that occurring in the atheroma.

In this context, while LDL has a proatherogenic role, HDL exerts an atheroprotective function [14]. The HDL cholesterol efflux capacity (CEC) is an estimate of the ability of HDL to promote cholesterol efflux from peripheral cells opposing foam cell formation, and it has emerged as a better predictor of CV risk compared to merely plasma HDL-cholesterol (HDL-c) levels, both in cross-sectional and prospective clinical studies [15,16,17,18,19].

On the other hand, the serum cholesterol loading capacity (CLC) is the ability of serum lipoproteins to induce cholesterol accumulation in macrophages, representing an index of serum atherogenicity. It is, in fact, raised in pathological conditions leading to a higher CV risk [20,21].

In this study, we present, for the first time, the existence of a relationship between alterations in serum lipoproteins functions and the presence of ATX in FH subjects. 

## 2. Results

### 2.1. Patients’ Characteristics

The subjects were stratified based on the absence (n = 16) or presence (n = 29) of objectively and/or ultrasonographically detectable ATX. The demographic and clinical features of the population are reported in Table 1. The two groups are homogenous in terms of age, sex and body mass index (BMI). Moreover, no significant differences were found in terms of high sensitive-C reactive protein (hs-CRP) serum levels. In subjects with ATX, the calculated DCLN score was significantly higher than those without ATX (*p* < 0.001), partly attributable to familiarity, with early atherosclerotic CVD found to be statistically more relevant in patients with xanthoma (51.7% vs. 18.8%; *p* = 0.03). The percentage of smokers was higher among subjects not presenting with ATX (50 % vs. 20.7 %; *p* = 0.04), while subjects did not differ in terms of presence of hypertension or diabetes mellitus. 

The two groups displayed no significant differences in terms of the plasma lipid profile (total cholesterol, HDL-c, LDL-c and triglycerides). Similarly, plasma levels of oxidized LDL and apolipoprotein B (apoB) did not differ between groups. 

The intensity and type of cholesterol-lowering therapy, mainly statins and ezetimibe, do not differ either in the two groups; only a small fraction of subjects was untreated, with equal distribution between subjects with or without ATX.

### 2.2. HDL Cholesterol Efflux Capacity (CEC)

**Figure 1:**
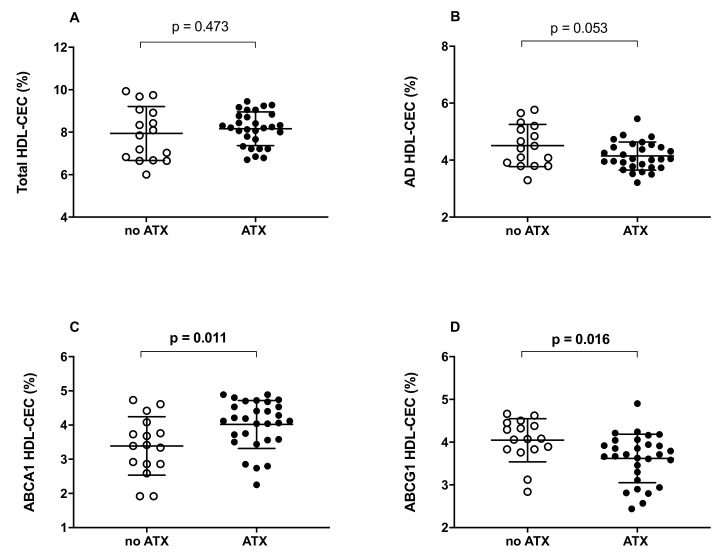
HDL cholesterol efflux capacity (CEC) in FH subjects not presenting or presenting ATX. (**A**): total HDL-CEC; (**B**): AD HDL-CEC; (**C**): ABCA1-mediated HDL-CEC; (**D**): ABCG1-mediated HDL-CEC. Each point of the graph represents the average percentage of triplicate analyses for each sample. The average of each group is represented by a horizontal, solid line. ○ No ATX: FH subjects not presenting ATX; ● ATX: FH subjects presenting ATX. Significant values are shown in bold.

In the patients’ cohort, we first evaluated the capacity of serum HDL to promote cholesterol efflux (HDL-CEC). Results are reported in Figure 1. The two groups of subjects did not show statistical differences in terms of total HDL-CEC (Figure 1A, *p* = 0.473). However, by individually analyzing the two contributions to total HDL-CEC, the efflux mediated by the aqueous diffusion (AD) process (AD HDL-CEC) and the efflux mediated by the ATP-binding cassette transporter A1 (ABCA1 HDL-CEC), we found a trend towards a reduction in AD HDL-CEC in FH patients presenting ATX compared to patients not presenting ATX (−8%; *p* = 0.053; Figure 1B). Regarding the ABCA1-mediated HDL-CEC, we found higher values in FH subjects presenting ATX compared to those without ATX (+18.6%; *p* = 0.011; Figure 1C). Conversely, the presence of ATX was associated with a significantly reduced HDL efflux capacity mediated by the ATP-binding cassette transporter G1 (ABCG1 HDL-CEC) (−11%; *p* = 0.016; Figure 1D). Similar results were obtained by stratifying subjects in subgroups according to the absence or presence of pharmacological treatment (data not shown).

### 2.3. Serum Cholesterol Loading Capacity (CLC)

**Figure 2:**
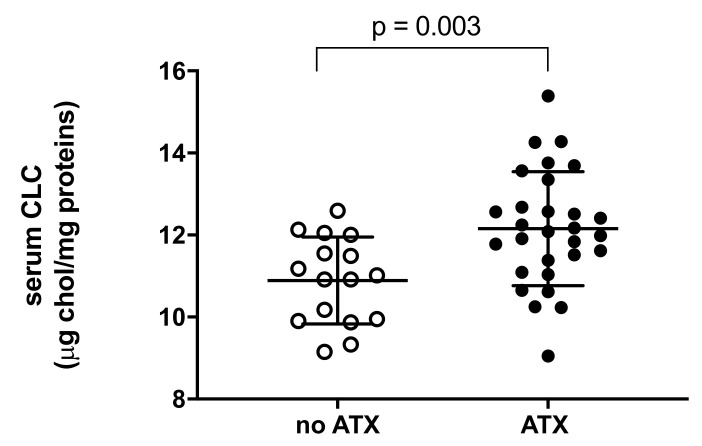
Serum cholesterol efflux capacity (CLC) in FH subjects not presenting or presenting ATX. Each point of the graph represents the average percentage of triplicate analyses for each sample. The average of each group is represented by a horizontal, solid line. ○ No ATX: FH subjects not presenting ATX; ● ATX: FH subjects presenting ATX.

As cell cholesterol content is the result of cholesterol efflux and influx, we evaluated whether the presence of ATX is associated with changes in the pro-atherogenic potential of the serum by measuring its cholesterol loading capacity (CLC) in macrophages. We found that serum CLC was significantly increased in patients with ATX compared to those without, showing an increment of 14% (*p* = 0.003; Figure 2).

**Figure 3:**
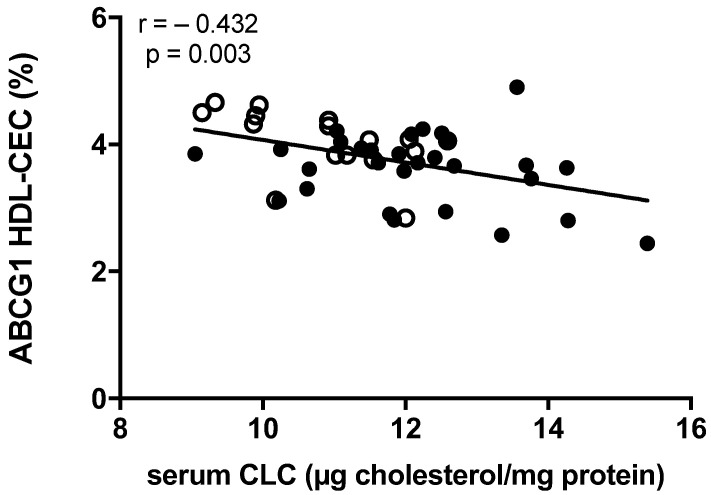
Correlation between ABCG1-mediated HDL-CEC and serum cholesterol loading capacity (CLC) in FH subjects. Pearson correlation coefficient was reported. ○: subjects not presenting ATX; ●: subjects presenting ATX.

Additionally, by exploring the relationships between serum lipoprotein function parameters, we found an inverse and robust correlation between serum CLC and the ABCG1-mediated CEC of HDL (Figure 3), while no associations were found between CLC and the other cholesterol efflux pathways (data not shown).

### 2.4. Correlation between Lipoprotein Functions and Achilles Tendon Thickness

We then explored the possible association between serum lipoprotein functions and the Achilles tendon thickness.

Concerning the relationship with HDL-CEC through the different pathways, AD and the ABCG1-mediated processes showed inverse and significant correlations with Achilles tendon thickness (Table 2). Achilles tendon thickness did not instead correlate with HDL-c levels (r = −0.255; *p* = 0.091).

**Figure 4:**
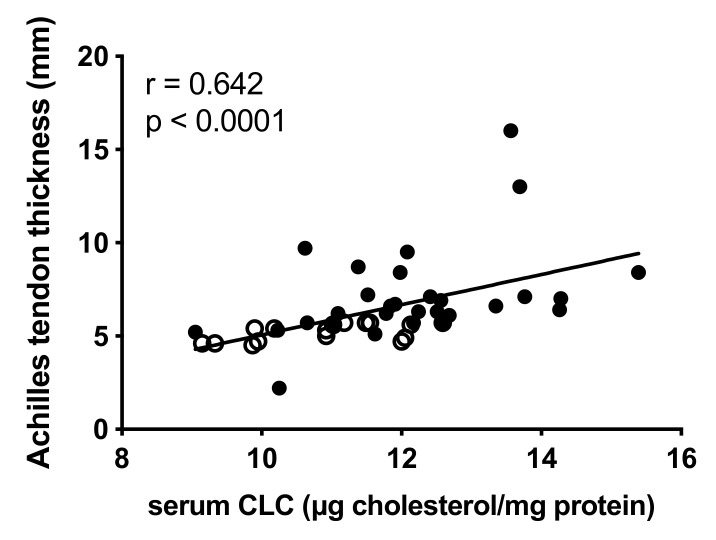
Correlation between serum cholesterol loading capacity (CLC) and Achilles tendon thickness in FH subjects. Spearman correlation coefficient was reported. ○: subjects not presenting ATX; ●: subjects presenting ATX.

Moreover, we observed a robust association between serum CLC and the Achilles tendon thickness (r = 0.642; *p* < 0.0001; Figure 4). Notably, Achilles tendon thickness was independent of plasma LDL-c (r = 0.194; *p* = 0.202), oxidized LDL (r = 0.099; *p* = 0.601) and apoB plasma levels (r = 0.141; *p* = 0.357). 

## 3. Discussion

Tendon xanthomas consist of accumulations of collagen and cholesterol ester-containing macrophages in the tendons [22], and their presence represents a pathognomonic sign of familial hypercholesterolemia (FH), a hereditary metabolic disorder characterized by high levels of LDL-c which favor the development of early ASCVD [2,9]. Achilles tendon xanthoma (ATX) plays a major role in the diagnostic definition of FH, leading the patient to early treatment of the disease, and its evaluation may provide a better cardiovascular risk assessment, being associated with premature cardiovascular disease (CVD) [7,8].

In this study, the serum lipoprotein functions (i.e., the capacity of high-density lipoprotein (HDL) to promote cell cholesterol efflux and serum capacity to promote cell cholesterol loading) were evaluated in FH patients to compare subjects presenting ATX with those not presenting ATX. This protocol allowed us to correlate, for the first time, lipoprotein functions with cholesterol accumulation in peripheral tissues. The two groups of patients were homogeneous in terms of age, BMI, lipid profile and lipid-lowering treatments, variables that might affect xanthoma development [13,23]. Importantly, the main finding of the present study was that the serum cholesterol loading capacity (CLC) was significantly raised in ATX-presenting patients as compared to patients without ATX.

Our findings clearly suggest that the presence of a serum with a high CLC might directly contribute to cholesterol accumulation within the tendons, promoting tendon thickening. This relationship is strengthened by the positive correlation between serum CLC and Achilles tendon thickness found in this study, again clearly indicating that a higher serum CLC is associated with cholesterol accumulation in peripheral tissues such as macrophages in Achilles tendons. On the other hand, LDL-c, apoB and oxidized LDL seem not to contribute to Achilles tendon thickness.

The pathophysiological process responsible for the formation of tendon xanthomas has significant similarities with that of pathogenesis and progression of atherosclerotic plaque, involving cholesterol accumulation in macrophages of arterial wall and foam cell formation [24]. Thus, the direct relationship between serum CLC and Achilles tendon thickness, reflecting an association with cholesterol accumulation in macrophages, may very likely also indicate a correlation with cholesterol accumulation in the atherosclerotic plaque of the arterial wall. Consistent with this conclusion, both the presence of xanthomas and high serum CLC are associated with a greater cardiovascular risk [20,25]. 

Although a higher level of total cholesterol and LDL-c is reported in patients with xanthoma than in those without xanthoma [25], in our study, LDL-c levels, ApoB and oxidized LDL were similar in the two groups of patients. In line with our data, other studies reported the absence of lipid level modifications in subjects with xanthoma [26,27]. This observation suggests that the overall ability of serum to deliver cholesterol to cells (CLC), rather than just the LDL-c concentrations, is relevant to ATX formation. This is consistent with the observation that not all FH subjects develop ATX despite high LDL-c levels [28] and sharing the same LDL receptor gene mutation [29].

Among LDL subclasses, the small dense particles have an increased ability to induce macrophage cholesterol accumulation [30,31]. In this respect, a case report by Mancuso and collaborators reported that the serum of a patient with ATX showed the presence of small and dense very low dense LDL (VLDL) and LDL. In this patient, an unusual quantity of conjugated dienes of arachidonic acid in the plasma and the LDLs was reported, which is present only in small traces in the control population [27]. These data suggest that, in patients with xanthoma, qualitative lipoprotein abnormalities may possibly explain the higher CLC that we observed. Additionally, the higher intracellular cholesterol content of macrophages exposed to sera from ATX patients in our model might also be related to some specific inflammatory factors present in serum that can directly modulate cellular cholesterol content [32,33]. Indeed, an increased concentration of tryptase, TNF-α, IL-8 and IL-6 in plasma from FH subjects with xanthoma as compared to subjects without xanthoma has been previously reported [34]. However, consistent with Nielsen et al. [35], in our cohort of patients, the presence of xanthoma was not associated with significant changes in the levels of hs-CRP, ruling out a possible role of plasma inflammatory factors in the observed increased CLC. 

Another important factor influencing macrophage cholesterol homeostasis is the capacity of HDL to interact with the membrane transporter ATP-binding cassette A1 (ABCA1) and G1 (ABCG1), thereby promoting cholesterol efflux and opposing LDL cholesterol loading. 

In our study, the ABCG1-mediated HDL capacity to promote cholesterol efflux (CEC) was lower in patients with xanthoma and inversely correlated with CLC in the entire population of our study. In addition, ABCG1-mediated HDL-CEC inversely correlated with Achilles tendon thickness. These results suggest that the increased CLC in ATX patients and its correlation with xanthoma formation may be, at least in part, explained by the reduced ABCG1-mediated HDL-CEC, according to the notion that serum CLC is the net result from all serum lipoprotein contribution. On the contrary, ABCA1-mediated HDL-CEC in patients with ATX raised as compared to no ATX FH patients and did not correlate with CLC, suggesting its minor role in CLC level and xanthoma formation.

The observed reduction of ABCG1-mediated CEC in ATX-presenting patients occurred despite no significant changes in HDL serum levels, consistent with the previously reported weak or absent relationship of this parameter with serum HDL-c levels [36,37]. Conversely, the capacity of HDL to interact with specific membrane cholesterol transporters depends on the maturation process they undergo in serum, which generates different HDL particle subclasses. The significantly lower ABCG1 HDL-CEC and the higher ABCA1 HDL-CEC found in patients presenting ATX suggests a shift of HDL particle distribution toward lipid-poor pre-β HDL, which interacts specifically with ABCA1 [15] with a consequent reduction of mature particles, with more affinity for the ABCG1-mediated pathway [38]. A similar impact of HDL remodeling on both ABCA1 and ABCG1 in parallel has been recently reported (https://doi.org/10.3389/fmolb.2022.925587, accessed on 27 June 2022).

The hypothesis of a defect in the maturation process of HDL might involve an alteration of the activity of the plasma enzymes responsible for HDL remodeling, such as lecithin-cholesterol acyltransferase (LCAT) and/or cholesteryl ester transfer protein (CETP) [39,40]. In this regard, increased apoA-I catabolism and the presence of smaller HDL particles in FH patients have been suggested to be the consequence of enhanced CETP activity along with reduced LCAT activity [41]. 

In addition, HDL compositional changes in terms of protein and lipid cargo may also be hypothesized [42]. For instance, elevated sphingomyelin (SM) and saturated fatty acid content, as well as an increase in cholesterol esters and triglyceride content, was observed in HDL_3_ particles isolated from FH patients [43]. 

In the present study, we also observed an inverse association between HDL-CEC via the aqueous diffusion (AD) process and ATX thickness, a result in line with what was observed by Ogura et al. [44]. However, unlike ABCG1 HDL-CEC, the AD-mediated HDL-CEC did not correlate with CLC, ruling out a significant role of this efflux pathway in the higher CLC found in our FH patients with xanthoma. Indeed, a reduced maturation of HDL has been observed in high-risk subjects [36].

Beyond favoring foam cell cholesterol accumulation, a defective ABCG1 HDL-CEC may contribute to xanthoma formation by affecting the inflammatory signaling in macrophages [45]. In this regard, in clinical studies, a specific association between ABCG1 HDL-CEC impairment and inflammation indexes has been reported [36,37,46]. In the context of tendon xanthoma formation, the drop of ABCG1 efflux observed might increase the inflammatory status of macrophages within the tendons, favoring foam cell formation. According to this hypothesis, macrophages from FH subjects with xanthoma showed a higher inflammatory status [11] and spontaneously released higher amounts of inflammatory cytokines compared to cells from FH patients without xanthoma [47]. 

The present study has some limitations mainly related to the small sample size of the analyzed cohort, even though it was sufficiently powered to support the overall changes in serum CLC and HDL-CEC between subjects with or without ATX. Second, the ultrasonographic cut-off established to distinguish tendons with xanthomas from those without is not standardized in the literature but rather derived by internal validation from previous analyses performed in the same population. However, many of the reported results were obtained from the entire sample by considering the tendon’s thickness.

An additional limitation may be the observational nature of the study. In this regard, it will be interesting in the future to add mechanistic insights to our findings by evaluating, for example, HDL metabolism as well as changes in HDL or LDL size, HDL remodeling enzyme activity and HDL/LDL lipid or protein composition that would explain the changes in serum lipoprotein functions that we observed in FH subjects presenting ATX.

In summary, for the first time, we reported an association between the presence of ATX in FH subjects with serum lipoprotein function derangement, namely a higher CLC. The increased CLC of sera from our patients with xanthoma is not related to changes in plasma lipids but rather to a reduction in the ABCG1 HDL-CEC. In conclusion, although the results need to be supported by wider studies, our observations provide evidence that lipoprotein dysfunctions are involved in tendon xanthoma physiopathology and, more in general, in cholesterol accumulation in peripheral tissues.

## 4. Materials and Methods

### 4.1. Study Population 

From January 2016 to September 2020, 349 hypercholesterolaemic subjects (mean age 43.1 ± 20.5; males 163, females 186) were evaluated at the Medical Department of the University of Padua for a clinical suspicion of FH based either on DLCN score or on physician’s judgment. Clinical and biochemical data were collected, and molecular analysis of the main FH-causing genes was performed within the project LIpid transPort disorders Italian GEnetic Network (LIPIGEN), an integrated network of Italian lipid clinics [48]. Finally, Achilles tendon ultrasonography was performed in all subjects at enrolment. All participants provided written informed consent.

We analyzed 45 patients (mean age 4.8 ± 15.6; males 16, females 29). FH causing mutations in the LDLR, apoB, Proprotein Convertase Subtilisin/Kexin type 9 (PCSK9) or Low-Density Lipoprotein Receptor Adaptor Protein 1 (*LDLRAP1*) genes were found in 78% (n = 35) of subjects. Furthermore, 4 patients had polygenic hypercholesterolaemia, and in the remaining 6, any pathogenic variant was found. The latter 6 cases had a definite clinical diagnosis of FH according to the DLCN, but no pathogenic variant was identified. They are likely carriers of yet-unidentified genetic mutations associated with the hypercholesterolemic phenotype; this finding is in line with the prevalence reported in published studies of unknown mutations associated with hypercholesterolemia, reported in about 20% of FH subjects [49,50].

### 4.2. Data Collection and Xanthoma Evaluation 

For each patient, we collected demographic and clinical data according to a standardized case report form. Specifically, we examined the familial and personal medical history of hypercholesterolemia and atherosclerosis ASCVD (i.e., acute coronary syndrome, angina, coronary intervention, stroke and peripheral arterial disease) for the clinical diagnosis of FH according to DCLN score. We also considered the other CVD risk factors (i.e., smoking habit, hypertension, diabetes) and any ongoing type of lipid-lowering treatment.

On the physical examination, we calculated the BMI. Then we searched the typical FH signs (tendon xanthomas and corneal arcus). Achilles tendon xanthoma (ATX) was clinically determined by one experienced observer as focal nodularities or if tendons were diffusely enlarged. The presence of ATX was also detected with bilateral Achilles tendon ultrasonography (Toshiba Aplio XV, linear probe 5–10 MHz) performed by a single trained operator. The patient lay in a prone position with his feet beyond the bed and flexed at 90°, and the physician acquired bilateral longitudinal and sagittal scans to detect the presence of xanthomas and measure anteroposterior tendon thickness at the point of maximum thickness; the maximum value obtained on both sides was considered for the analyses. ATX was defined as either the presence of a single or multiple focal hypoechoic lesions within the tendon or a widespread loss of tendon fibrillary structure or a tendon’s maximum anteroposterior diameter thickness > 6.1 mm. Subjects with a history of inflammatory or degenerative tendinopathy or with previously reported Achilles tendon traumatic injury were excluded from the study.

### 4.3. Total Cholesterol, HDL Cholesterol, Triglycerides and hs-CRP Evaluation

Total cholesterol, HDL-c and triglycerides were measured using standard enzymatic methods; the LDL-c levels were calculated according to the Friedwald formula. If not available, the pre-treatment LDL-c value was estimated by multiplying LDL-c during therapy for a correction factor established by dose and type of current lipid-lowering treatment [51]. Inter- and intra-assay variation coefficients (CV) for total cholesterol measurement were 2.78% and 0.78% respectively; for triglycerides measurement, these values were 1.64% and 1.80%, respectively. 

High-sensitive C reactive protein (hs-CRP) plasma concentrations were evaluated by a commercial ELISA kit (apDia, Turnhout, Belgium, cod. 740011) upon 1:1000 sample dilution, as previously described [52]. Sample concentrations were obtained by generating a quadratic polynomial regression (GraphPad Prism v8.2.1, San Diego, CA, USA). The minimal detectable concentration is 0.02 μg/ml. Inter- and intra-assay variation coefficients (CV) were 6.1 ± 0.29% and 5.1 ± 1.56%, respectively.

### 4.4. HDL Cholesterol Efflux Capacity (CEC)

HDL’s capacity to promote cholesterol efflux (CEC) through the main pathways was evaluated in patients’ HDL fraction by a standardized and widely used radioisotopic cell-based technique [53]. For HDL fraction isolation, the apoB-containing lipoproteins were precipitated from whole serum with a solution of polyethylene glycol [54]. This procedure, which provides biological samples containing only HDLs, is comparable to HDL isolation through ultracentrifugation for the CEC study [55].

To prevent remodeling of the lipoproteins, sera were slowly thawed in ice immediately prior to this procedure.

### 4.5. Total, Aqueous Diffusion and ABCA1 HDL-CEC

Total HDL-CEC and its major constituents, the efflux through aqueous diffusion (AD) process (AD HDL-CEC) and thorough the ATP binding cassette transporter A1 (ABCA1 HDL-CEC), were performed on the J774 murine macrophages cell line. In particular, in basal conditions, J774 were used to evaluate AD, whereas, to measure total HDL-CEC, J774 were incubated with a cAMP analogue (cpt-cAMP 0.3 mM; Sigma-Aldrich, Saint Louis, MO, USA), inducing ABCA1 expression [56]. The ABCA1-mediated efflux was determined as the difference between total HDL-CEC and AD HDL-CEC. For the seeding period, J774 macrophages were plated in 10% fetal calf serum (FCS) containing DMEM (both FCS and DMEM from Euroclone, Milano, Italy) supplemented with antibiotics (penicillin–streptomycin from Thermo Fisher Scientific, Waltham, MA, USA). For the labeling period, cells were incubated for 24 h with [1,2-^3^H] cholesterol (PerkinElmer, Waltham, MA, USA) at 2 μCi/mL, and, to prevent accumulation of cholesteryl esters, in the presence of 2 μg/mL of an inhibitor of the cholesterol esterifying enzyme acyl-coenzyme A: cholesterol acyltransferase (Sandoz 58035; Sigma-Aldrich, Saint Louis, MO, USA). After 18 h of equilibration in the absence or presence of cAMP analogue in 0.2% bovine serum albumin-containing medium (BSA from Sigma-Aldrich, Saint Louis, MO, USA), J774 were incubated for 4 h with 2% (*v*/*v*) of HDL fraction from FH patients with and without ATX. HDL-CEC was given as a percentage: the amount of radiolabeled cholesterol released into the medium was corrected for the total radioactivity incorporated by cells. As internal controls, the HDL fraction of a standard serum, obtained from a pool of normolipidemic subjects, and lipid-free human apolipoprotein A-I (Sigma-Aldrich, Saint Louis, MO, USA) were tested together with subjects’ HDL fraction in each assay. To correct for the inter-assay variability, the HDL-CEC percentage in these conditions was used to normalize the different experiments. Intra-assay CV for HDL-CEC assays was <10%.

### 4.6. ABCG1-Mediated HDL-CEC 

HDL CEC through the ATP binding cassette transporter G1 (ABCG1) (ABCG1 HDL-CEC) was measured on Chinese hamster ovary (CHO) cells transfected and not transfected with the human *ABCG1* gene. The difference between HDL-CEC evaluated in ABCG1-transfected cells and in non-transfected cells represents the specific ABCG1 contribution. For the seeding period, CHO cells were plated in 10% FCS containing Ham’s F-12 (from Euroclone, Milano, Italy) in the presence of antibiotics (zeocin and penicillin–streptomycin from Thermo Fisher Scientific, Waltham, MA, USA). Cells were labelled for 24 h with [1,2-^3^H] cholesterol at 1 μCi/mL. Then they underwent an equilibration period for 90 min in a 0.2% BSA-containing medium. Cells were successively incubated for 6 h with the 1% (*v*/*v*) of the HDL fraction from FH patients with and without ATX. HDL-CEC was given as a percentage: the amount of radiolabeled cholesterol released into the medium was corrected for the total radioactivity incorporated by cells. As internal controls, the HDL fraction of a standard serum, obtained from a pool of normolipidemic subjects, and isolated human HDLs were tested together with subjects’ HDL fraction in each assay. Human HDLs (d 1.063–1.21 g/mL) were isolated and purified from the plasma of healthy volunteers not affected by FH by sequential ultracentrifugation. 

To correct for the inter-assay variability, the HDL-CEC percentage of these conditions was used to normalize the different experiments. Intra-assay CV for HDL-CEC assays was <10%.

### 4.7. Serum Cholesterol Loading Capacity (CLC)

As for CEC, to prevent lipoprotein remodeling, sera were slowly thawed in ice immediately prior CLC measurement. CLC was evaluated with a fluorometric technique on human monocyte-derived THP-1 macrophages [57]. Human THP-1 monocytes were seeded in 10% FCS containing RPMI (both from Euroclone, Milano, Italy) supplemented with antibiotics (penicillin–streptomycin from Thermo Fisher Scientific, Waltham, MA, USA). To allow monocyte differentiation into macrophages, cells were plated for 72 h with 100 ng/mL phorbol 12-myristate 13-acetate (Sigma-Aldrich, Saint Louis, MO, USA). Cells were then exposed for 24 h to 5% human lipoprotein-deficient serum (Sigma-Aldrich, Saint Louis, MO, USA) and, successively, incubated with 10% (*v*/*v*) of whole serum from FH patients with and without ATX for 24 h. After that, cell monolayers were lysed in a solution of 1% sodium cholate (Sigma-Aldrich, Saint Louis, MO, USA) and 10 U/mL DNase (Sigma-Aldrich, Saint Louis, MO, USA). On cell lysates, cholesterol was measured fluorometrically using the Amplex Red Cholesterol Assay Kit (Molecular Probes, Eugene, OR, USA) following the manufacturer’s instructions, and cell protein content was evaluated by the bicinchoninic acid assay (Thermo Fisher Scientific, Waltham, MA, USA). CLC was indicated as micrograms of cholesterol on milligram of protein. As an internal control, sera obtained from pools of normolipidemic and hypercholesterolemic subjects were tested together with whole subjects’ serum samples in each assay to check for adequate cell responsiveness. To correct for inter-assay variability, the CLC values of these conditions were used to normalize the different experiments. Intra-assay CV for the CLC assays was <10%.

### 4.8. Statistical Analysis 

For a priori sample size estimation, G*Power software (Düsseldorf, Germany) was used. Relying on data from a previous study [44] that compared FH patients with and without atherosclerotic cardiovascular disease (ASCVD) and using HDL-CEC as the primary endpoint, a sample size of at least 15 individuals per group was required, with an alpha of 0.05 and power of 80%. 

Statistical analyses were executed using GraphPad Prism version 7.00 (GraphPad Software, San Diego, CA, USA) and IBM SPSS software. Each sample was run in triplicate. Data are given as mean ± SD for parameters with normal distribution or as median with interquartile (IQR) range (25th to 75th percentile) for parameters with skewed distribution. Normality of distribution was assessed using the D’Agostino and Pearson normality test. Differences between FH patients with and without ATX were evaluated using the unpaired two-tailed Student’s t-test for parameters with normal distribution or the Mann–Whitney test for parameters with skewed distribution. 

Correlation analyses were assessed to highlight the relationship between parameters by using univariate logistic regression. Pearson correlation coefficients were reported for data with normal distribution, and Spearman correlation coefficients were reported for data with skewed distribution. The statistical significance was identified as *p* < 0.05.

## Figures and Tables

**Table 1:** General characteristics of the study population.

Characteristics	Xanthoma		*p* Value
	None (n = 16)	Present (n = 29)	
**Age**—years	42.1 ± 18.6	46.3 ± 13.9	n.s.
**Male**—n. (%)	5 (31.2%)	11 (37.9%)	n.s.
**BMI**—Kg/m^2^	24.2 ± 5.3	25.9 ± 5.8	n.s.
**hs-CRP**—µg/mL	0.54 (1.7)	0.25 (0.94)	n.s.
**DLCN score**	6.4 ± 3.1	11.0 ± 5.0	<0.001
**Cardiovascular risk factors**—n. (%)			
Smoking	8 (50.0%)	6 (20.7%)	0.04
Arterial Hypertension	1 (6.3%)	3 (10.3%)	n.s.
Diabetes mellitus	0	0	n.s.
ASCVD familiarity	3 (18.8%)	15 (51.7%)	0.03
Early cardiovascular events—n. (%)	3 (18.8%)	3 (10.3%)	n.s.
**Lipid profile**—mg/dL			
Total Cholesterol	223.8 ± 66.8	260.4 ± 80.7	n.s.
HDL Cholesterol	61.2 ± 16.6	55.9 ± 14.0	n.s.
LDL Cholesterol	141.3 ± 57.2	180.1 ± 77.0	n.s.
Triglyceride	106.9 ± 49.0	124.5 ± 77.1	n.s.
Oxidized LDL	56.3 ± 13.7	74.4 ± 34.3	n.s
apoB	109.7 ± 26.3	131.5 ± 44.2	n.s.	
**Hypolipemic therapy in progress**—n. (%)			
Statin	11 (68.6%)	16 (55.2%)	n.s.
Ezetimibe	4 (25.0%)	13 (44.8%)	n.s.
None	5 (31.2%)	13 (44.8%)	n.s.

ASCVD: atherosclerotic cardiovascular disease; BMI: body mass index; DLCN score: Dutch Lipid Clinic Network score; HDL: high-density lipoproteins; hs-CRP: high sensitivity C-reactive protein; IQR: interquartile range; LDL: low-density lipoproteins; med: median; n.s.: not significant; SD: standard deviation. Normally distributed continuous parameters were presented as mean ± SD, and skewed continuous parameters were expressed as the median and interquartile range (defined as 25th percentile to 75th percentile).

**Table 2:** Correlation between HDL cholesterol efflux capacity (CEC) and Achilles tendon thickness.

HDL-CEC Pathways	r	*p* Value
Total HDL-CEC	−0.104	0.497
AD HDL-CEC	**−0.342**	**0.021**
ABCA1 HDL-CEC	0.186	0.221
ABCG1 HDL-CEC	**−0.367**	**0.013**

Correlation analyses were performed to highlight the relationship between parameters, and the Spearman correlation coefficients were reported. Significant associations are shown in bold.

## Data Availability

The authors declare that the data generated and analyzed during this study are included in this article. In addition, datasets generated and/or analyzed during the current study are available from the corresponding author on reasonable request.
